# Granulocyte dynamics: a key player in the immune priming effects of crickets

**DOI:** 10.3389/fimmu.2024.1383498

**Published:** 2024-05-17

**Authors:** Youngwoo Cho, Saeyoull Cho

**Affiliations:** ^1^ Department of Plant Medicine, College of Agriculture and Life Science, Kangwon National University, Chuncheon, Republic of Korea; ^2^ Department of Interdisciplinary Program in Smart Agriculture, College of Agriculture and Life Science, Kangwon National University, Chuncheon, Republic of Korea

**Keywords:** immune priming, granulocytes, survival rates, *Bacillus thuringiensis*, gene expression analysis, invertebrate immunity

## Abstract

This study investigates immune priming effects associated with granulocytes in crickets through a comprehensive analysis. Kaplan-Meier survival analysis reveals a significant contrast in survival rates, with the heat-killed *Bacillus thuringiensis* (*Bt*)-primed group exhibiting an impressive ~80% survival rate compared to the PBS buffer-primed group with only ~10% survival 60 hours post live *Bt* infection. Hemocyte analysis underscores elevated hemocyte counts, particularly in granulocytes of the killed *Bt*-primed group, suggesting a correlation between the heat-killed *Bt* priming and heightened immune activation. Microscopy techniques further explore granulocyte morphology, unveiling distinctive immune responses in the killed *Bt*-primed group characterized by prolonged immune activation, heightened granulocyte activity, phagocytosis, and extracellular trap formation, contributing to enhanced survival rates. In particular, after 24 hours of injecting live *Bt*, most granulocytes in the PBS buffer-primed group exhibited extracellular DNA trap cell death (ETosis), while in the killed *Bt*-primed group, the majority of granulocytes were observed to maintain highly activated extracellular traps, sustaining the immune response. Gene expression analysis supports these findings, revealing differential regulation of immune-related genes such as antibacterial humoral response, detection of bacterial lipopeptides, and cellular response to bacteria lipopeptides. Additionally, the heat-killed *Bt*-primed group, the heat-killed *E. coli*-primed group, and the PBS-primed group were re-injected with live *Bt* 2 and 9 days post priming. Two days later, only the PBS-primed group displayed low survival rates. After injecting live *Bt* 9 days later, the heat-killed *E. coli*-primed group surprisingly showed a similarly low survival rate, while the heat-killed *Bt*-primed group exhibited a high survival rate of ~60% after 60 hours, with actively moving and healthy crickets. In conclusion, this research provides valuable insights into both short-term and long-term immune priming effects in crickets, contributing to our understanding of invertebrate immunity with potential applications in public health.

## 1 Introduction

When invertebrates and vertebrates are invaded by bacteria, mold, parasites, they manage to survive by effectively eliminating these invaders through sophisticated immune responses. In other words, the majority of organisms generate immune responses to eliminate diverse pathogens, ultimately aiming for survival and reproduction. However, the immune responses observed in the two taxa exhibit some differences. Vertebrate utilize both adaptive and innate immunity to defend against pathogens (1). In vertebrates, adaptive immunity, characterized by T cells, B cells, and dendritic cells, provides antigen-specific responses and maintains long-term immunological memory (2). On the contrary, insects are generally recognized for primarily relying on innate immunity. They eliminate foreign substances by swiftly generating a non-specific immune response, lacking immune memory, against various pathogens that have infiltrated the body (3).

The innate immune response of insects consists of the humoral immune system and the cellular immune system (4). The humoral immune system involves overexpressed antimicrobial peptides (AMPs) directly contacting the cell membrane of pathogens, forming holes to eliminate them. In addition, the cellular immune system of insects is an immune response in which major immune hemocytes recognize invading pathogens, contact them, and eliminate pathogens using phagocytosis, encapsulation, and nodulation. In general, insect hemocytes are recognized for their inability to remember invading pathogens or produce antibodies. In other words, insects do not have cells that play the same role as mammalian B and T lymphocytes.

Although the types of hemocytes in the hemocoel of insects differ depending on the insect species, usually, there are seven types of hemocytes: prohemocyte, granulocyte, plasmatocyte, oenocytoids, spherulocyte, adipohemocyte, and coagulocytes. The functions of these hemocytes vary slightly depending on the species, but granulocytes and plasmatocytes are reported to be the main immune cells in many species of insects (5, 6). However, it is reported that insect granulocyte and plasmatocytes do not have the same function as mammalian B and T lymphocytes, and play the same role as neutrophil, natural killer cells, and macrophage, which are responsible for mammalian innate immunity. Therefore, it is evident that a notable disparity exists in the functionality of immune cells between vertebrates and invertebrates. Nonetheless, numerous parallels have also been documented. For instance, mammalian neutrophils demonstrate heightened activity in forming extracellular traps upon encountering substantial pathogen loads. Moreover, the occurrence of ETosis, characterized by cell rupture with extracellular traps and death induced by the expression of genes associated with reactive oxygen species (ROS), is widely acknowledged (7). Notably, this phenomenon has recently been observed in the phagocytes of blue mussels, sea anemones, and crabs, suggesting its prevalence across invertebrates (8). Furthermore, ETosis has been identified in the phagocytes of *Galleria mellonella*, an insect species, although investigations in this domain are still in their nascent stages (9).

Immune priming occurs during the initial exposure to an antigen (which can be a pathogen or a component of a pathogen), and it involves the activation and expansion of antigen-specific B and T lymphocyte. While immune priming is a critical aspect of the adaptive immune response, the term is often used more broadly to describe the overall preparation and enhancement of the immune system’s ability to respond to specific antigens. It plays a central role in vaccination strategies, where controlled exposure to a harmless form of a pathogen induces immune priming and establishes immunological memory. In recent years, there has been growing interest and research on the presence of immune memory or immune priming in invertebrates, including insects. The Immune priming effects has also been proven in insects in a variety of ways. For example, it is proven by injecting a minimum amount of pathogens that lost their activity (non-pathogenicity) into species *in vivo*, then re-injecting active pathogens, and examining survival rates and expression levels of immune-related genes such as AMPs compared to the control group (10). These immune priming effects can be observed in *Crassostrea gigas*, *Anopheles gambiae*, *Drosophila melanogaster*, *Manduca sexta, Apis mellifera*, *Bombus terrestris*, *Galleria mellonella*, *Tribolium castaneu*m (11–15). Indeed, research in *A. gambiae*, has provided intriguing insights into the potential relationship between rapid differentiation of hemocytes and immune memory. Additionally, the role of microorganisms in the gut (intestinal microbiota) has emerged as a significant factor in immune priming (16). These examples collectively demonstrate that immune priming is a widespread phenomenon in the animal kingdom, extending beyond traditional models of immune memory observed in vertebrates. While the research in this area is ongoing, and the mechanisms of potential immune memory in invertebrates are not yet fully understood, these findings challenge the traditional view of invertebrates lacking adaptive immune memory. Further investigations are needed to unravel the underlying cellular and molecular mechanisms and to determine the extent and specificity of immune memory in different invertebrate species, including insects (17–20).

This study investigated the effects of short-term or long-term priming in connection with immune cell activation in *Gryllus bimaculatus*. In the past, we conducted cellular immunity studies with various insects (21–24). In particular, we reported that cricket granulocytes perform very active cellular immune responses (phagocytosis, encapsulation, and nodulation) by forming pod-, fan-, and web-structures on their cell membrane when they come into contact with invading pathogens (24). Based on these researches, we induced immune priming using non-pathogenic substances and reinjected active pathogens to measure survival rate, immune responses of granulocytes, and lysosomes activation among immune priming challenged groups. Additionally, we identified and compared the expression of immune-related genes between groups through high-throughput RNA-sequencing.

## 2 Materials and methods

### 2.1 Experimental design

**Figure 1:**
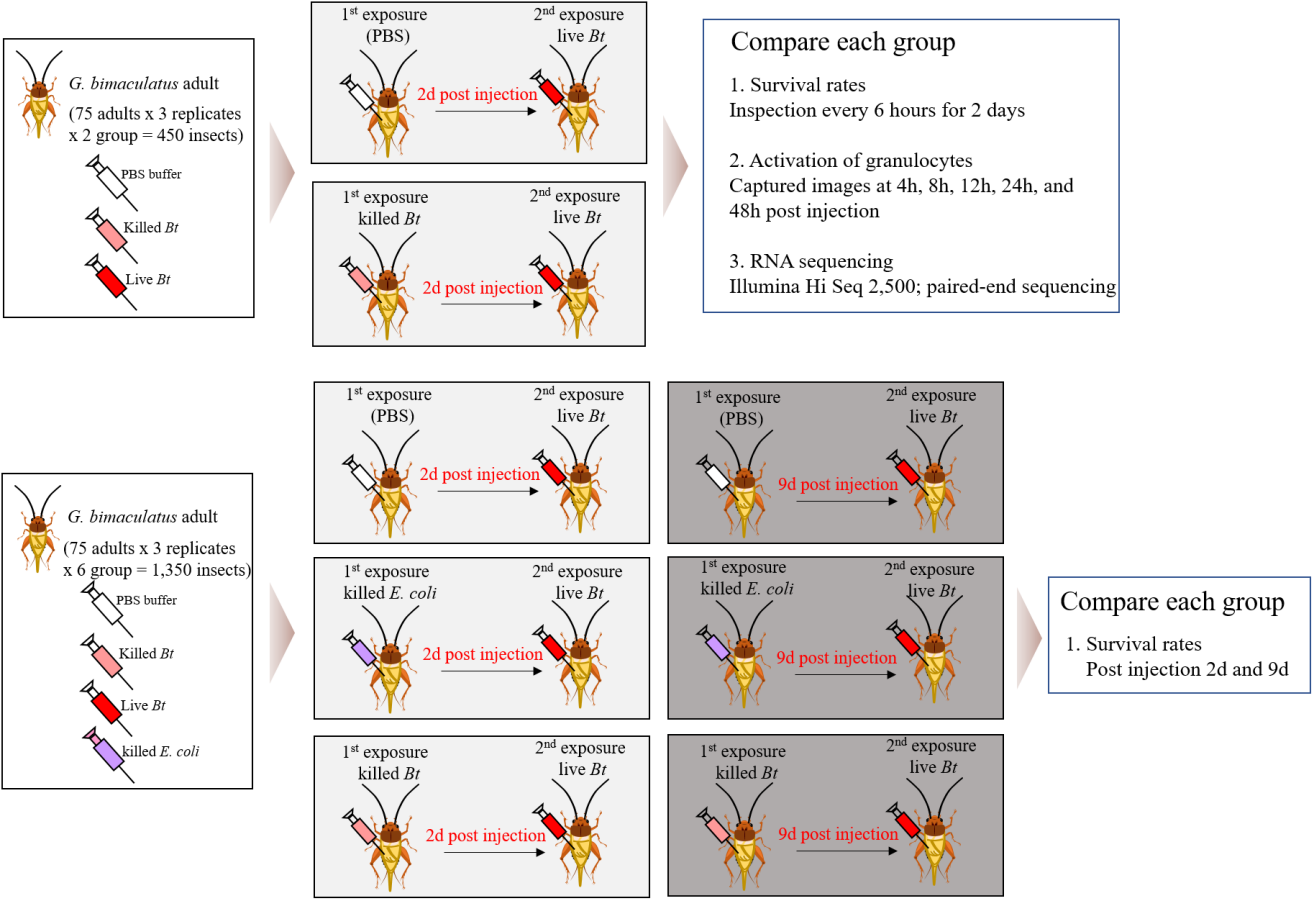
Comprehensive analysis of immune responses in crickets through varied pathogen priming and subsequent exposures. The crickets were divided into eight groups and conducted the experiments with 75 crickets per group, 3 repetitions (8x75x3 = 1,800 crickets). Immune priming was induced using two groups, the PBS-primed group and the heat-killed *Bacillus thuringiensis* (*Bt*)-primed group. Two days later, live *Bt* was injected again to create two groups. Subsequently, the immune priming effect was assessed by examining survival rates, the immune activation of granulocytes, and conducting RNA-Seq analysis for both the groups. Next, three groups were primed with PBS, heat-killed *Escherichia coli* (*E*. *coli*), and heat-killed *Bt*. After 2 days, live *Bt* was injected into all groups, and survival rates were examined. Similarly, 9 days later, we injected live *Bt* into each primed group and assessed survival rates.

We divided 1,800 crickets into 8 groups and conducted the experiments with 75 crickets per group, 3 repetitions (8x75x3 = 1,800 crickets) (**Figure 1**). In this study, immune priming was induced using two groups, the PBS-primed group and the heat-killed *Bacillus thuringiensis* (*Bt*)-primed group. Two days later, live *Bt* was injected again to create two groups (**Figure 1**). Subsequently, the immune priming effect was assessed by examining survival rates, the immune activation of granulocytes, and conducting RNA-Seq analysis for both the groups. Next, three groups were primed with PBS, heat-killed *Escherichia coli* (*E*. *coli*), and heat-killed *Bt*. After 2 days, live *Bt* was injected into all groups, and survival rates were examined. Similarly, 9 days later, we injected live *Bt* into each primed group and assessed survival rates.

### 2.2 Experimental animals and husbandry

We collected 150 crickets from basement of a building or under rocks or log debris in mountain, mated them, and collected eggs. Female laid an average of 90 ± 10 eggs, and hatched young were reared in a sterilized incubator (MIR-553, Sanyo Electric Biomedical, Japan; 25 ± 1°C, 40 ± 10%, 16h light:8h dark) maintain a constant environment. We used second- and third-generation adults as experiment insects. We provided sterilized food (Chinese cabbage and wheat husk purchased from Milwormnara Ltd., Korea) and always maintained cleanliness. Healthy crickets consistently exhibit activity through antennae movement, while sick or weak crickets demonstrate diminished antennae mobility and a notable decline in overall activity levels. Therefore, when such crickets are found in a breeding cage, we immediately remove them.

### 2.3 Bacterial preparation and CFU determination

The bacteria strains utilized in in this study mentioned were *Bacillus thuringiensis* (*Bt*) strain KACC 12061 and *Escherichia coli* (*E. coli*) strain KACC16628, obtained from the Korean Agricultural Culture Collection (KACC). Briefly, the *B. thuringiensis* and *E. coli* strains originated from a single frozen glycerol stock, which was streaked on Luria-Bertani Broth plates (25 g.L^-1^ of LB base, Invitrogen 12795–027 supplemented with 15 g.L^-1^ of agar-agar, Fisher BioReagents, BP1423–2) and NA Broth (Beef extract 3gL, Peptone 5gL, Difco/BD, 234000) plates at 37°C and 25°C overnight. Subsequently, 100 µL of the *E. coli* culture was inoculated into 5 mL of fresh LB broth and incubated under the same conditions until reaching an optical density (OD) of 1.3 at 600 nm, as determined by an Ultrospec 10 (Amersham Biosciences), corresponding to 1.04x10^9^ cfu/mL. The bacterial pellet obtained by centrifuging 5 mL of the culture (2 minutes, 4°C, 13,000 g) was resuspended in 1 mL of PBS buffer to achieve an OD of 1.3 for injection. Similarly, 100 µL of the *B. thuringiensis* culture was inoculated into 3 mL of fresh NA broth and incubated under the same conditions until reaching an OD of 1.3 at 600 nm, as determined by an Ultrospec 10 (Amersham Biosciences), corresponding to 1x10^9^ cfu/mL. After centrifugation of 5 mL of the culture (2 min, 4°C, 13,000 g), the bacterial pellet was resuspended in 1 mL of PBS buffer to achieve an OD of 1.3 for injection. The LD_50_ (Lethal Dose, 50%) value was determined at an OD of 1.3 for *G. bimaculatus* and measured over a 96-hour period, with a dosage of 10 µL (1x10^7^ cfu/insect for *Bt* and 1.04x10^7^ cfu/insect for *E*. *coli*).

### 2.4 Priming and sample collection

All crickets were selected 24 hours after molting from young to adult. Additionally, we removed inactive or unhealthy individuals (less than ~2%) after the first injection and used only healthy individuals that were very active and feeding for the injection of live *Bt*. In all groups, the crickets were almost identical in age (24 hours after adult molting), health (active movement and no infection), average size (~25 ± 5 mm per cricket), and average weight (~1.1 ± 0.5g per cricket). Immune priming was performed by diluting the OD 1.3 obtained from the LD_50_ value at a ratio of 1:999, and 2μl was inoculated (2x10^3^ cfu/insect for *Bt* and 2.08x10^3^ cfu/insect for *E. coli*). The reason for this is similar to preventive vaccination, where we aimed to ensure that there was no impact on survival due to the preventive inoculation. Therefore, we obtained values over the entire observation period of 96 hours where there was no difference between the control group and the survival group. Next, 48 hours after the immune cycle of crickets, all individuals were injected with 10μl of live pathogen at the concentration described in material and method section 2.3. Subsequently, survival analysis and microscopic examinations were conducted at different time intervals. For injection, the crickets were sterilized with 70% alcohol paper, and a finely pulled glass needle was shallowly inserted into the abdomen (Haematocrit capillaries, Sigma-Aldrich) (17, 18). The experiments were repeated three times independently.

### 2.5 Hemocyte count analysis

Change in hemocyte count were conducted for group A-1 and B-1. Hemolymph was collected from each group by extracting 50 µL from each cricket. Subsequently, a total of 500 µL of hemolymph from 10 crickets was placed into a 1.5 mL microcentrifuge tube (Bioneer Ltd., Korea) containing 500 µL of coagulant solution (98 mM NaOH, 186 mM NaCl, 17 mM EDTA, and 41 mM citric acid, pH 4.5), followed by centrifugation (10 min at 4°C) to remove plasma (Beckman Inc., USA). Hemocytes (pellets) were washed three times with 1mL of sterile PBS buffer. To count total hemocyte (THC), we placed hemocytes on sterile disposal hemocyte meter slides (Ncubauer Improve, iNCYTO C-Chip DHC-N01; 10µL) and count them in four squares using a light microscope (Leica^®^ DMI 3000M; 40x objectives) and calculated as absolute numbers. The hemocyte count were calculated at 0, 12, 24, and 48 h post-infection.

### 2.6 Granulocyte immune activation, lysosome staining, and FACS analysis

For analysis using an optical microscope (Leica^®^ DM2500 and Leica^®^ DMI 3000B), 1,000 hemocytes per group were photographed and hemocytes image database for each group was constructed (LMD application software version 6.1, 2048x1536 pixels). We used this image data to classify hemocytes by size and shape (21–24). To analyze lysosome activation in granulocytes, we stained the hemocytes collected from each group using the acid affinity dye LysoTracker Red (7.5nM, Molecular Probes; Thermo Scientific, Waltham, USA) at room temperature for 30min, washed three times with PBS, and analyzed by a Leica DMI 3000B fluorescent microscope and flow cytometry (BD™ FACS Canto; BD Bioscience, San Jose, CA) (24). Red fluorescence was detected by channel FL3 (610/20 band-pass), 10,000 hemocytes were read per sample, and statistical processing was performed using BD Bioscience FASC Diva software (24). We provided the flow cytometry gating strategy as a **Supplementary File** (**Supplementary File S1**). For scanning electron microscopy (SEM), hemocytes for each group were sedimented on Thermanox™ 125 coverslip coated with 0.1% poly-L-lysine (Thermo Fisher Scientific, Waltham, USA) for 3 days. Hemocytes attached to the cover slip were fixed using sodium cacodylate buffer (Na (CH3)_2_AsO_2_, 0.1M, pH 6.5; 50mM isopropanol 15%, Sigma-Aldrich) containing 2.5% glutaraldehyde. The coverslip was fixed again with 1% osmium tetroxide (OsO_4_) and were dehydrated by gradual immersion in ethanol (70%~100%) and subjected to critical point drying (Leica^®^ EM CPD030). The coverslip was sputtered with gold and finally observed with an SEM Leo 420 scanning electron microscope (LEO Electron Microscopy Ltd., Cambridge, UK). For transmission electron microscope (TEM), hemocytes were fixed with 1% osmium tetroxide (OsO_4_) and then the dehydrated using ethanol (70%~100%) and 100% propylene oxide. Hemocytes were embedded in araldite resin (Epon 812, Japan) and polymerization reaction was performed at 60°C for 48 hours. Hemocytes were ultrathin sectioned using an ultramicrotome (Pabisch Top Ultra 150) and collected on a 200mesh nickel grid. Ultrathin sections were stained with uranyl acetate, precipitated in citrate for 5min, and observed using a JEX-1230 transmission electron microscope (JEOL Ltd, Japan).

### 2.7 Fatbodies and RNA extraction

To perform RNA sequencing, we extracted fatbodies from group A, B, A-1, and B-1. We selected 10 crickets from each group and prepared them in one tube for each group. Since each group was repeated 3 times, fatbodies were extracted from a total of 30 crickets and prepared in 3 tubes per group (12 tubes in total). Total RNA was extracted using the RNeasy Plus Mini Kit (Qiagen, USA) (all operations were performed using sterile spaces and tolls to minimize contamination). To briefly summarize the procedures, 100mg of fat bodies were mixed with 1ml of TRIzol reagent (Thermo Fisher Scientific, Waltham, USA). Plastic beads of 0.6 mm, 2 mm, and 7 mm were added to the mixture, and it was homogenized for 10 minutes with a vortex (DAIHAN Ltd., Korea). The sample were centrifuged at 14,000 rpm for 3 min at 4°C and 1mL of supernatant was extracted. The extracted supernatant was mixed with 200 µL chloroform and reacted at 4°C for 20 min (Thermo Fisher Scientific, Waltham, USA). The sample were centrifuged at 14,000 rpm for 3 min at 4°C for 15min and then the supernatant (~600 µL) was loaded into a gDNA elimination column to remove gDNA (Thermo Fisher Scientific, Waltham, USA). The sample was centrifuged at 14,000 rpm for 1 min at 4°C and then total RNA was precipitated by adding 600 µL of cold isopropanol/70% ethanol (maintained at -20°C, Sigma-Aldrich, USA). The sample was centrifuged at 14,000 rpm for 1min at 4°C using RNeasy Plus Mini column, mixed with ~700 µL of RW1 buffer (10mM Tris-Hcl, 1mM EDTA, 50mM NaCl, 0.5% Tween-20, pH 8.5, Sigma-Aldrich, USA), and centrifuged at 14,000rpm for 1min. Finally, the sample was mixed with 500 RPE buffer (10mM Tris-Hcl, 80% ethanol, 0.5mM EDTA pH 7.5), centrifuged, and total RNA was extracted by placing ~50 µL RNeasy-free water in a new sterilized tube (Sigma-Aldrich, USA).

### 2.8 RNA sequencing, *de novo* transcriptome assembly, and annotation

The cDNA library was generated from the total RNA, and paired-end sequencing was performed using the Illumina RNA-seq technology (Macrogen Ltd, Korea). Raw sequencing data was preprocessed using integrated software for adapter and quality trimming, Trim Galore v0.6.5. Reads with a Phred33 score of less than 20 and a length of less than 20 base pairs were filtered out, and the standard Illumina adapter sequence was trimmed if found. *De novo* full-length transcriptome reconstruction was performed by a software to reconstruct full-length transcriptome without a reference genome, Trinity v2.15.1, and ORF (open reading frame) prediction was performed by software to identified candidate coding region from *de novo* transcriptome assembly, TransDecoder v5.5.0 (25). Predicted ORFs were assessed using homology search (BLAST), protein domain identification (PFAM), gene ontology assignment, and functional classification (e.g., eggnog) of sequence data using software to annotate *de novo* transcriptome assembly, Trinotata v4.0.2 (26). Quantification of gene expression was performed using software to quantify expression of genes and transcripts from transcriptomic data, Salmon v1.10.2 (27).

### 2.9 Transcriptomic analysis

The gene expression result was normalized and transformed using a function provided by DESeq2 v1.36.0, Variance Stabilizing Transformation (VST). Principal Component Analysis (PCA) was conducted on the transformed gene expression data using the prcomp function from the R core package, stats v3.6.2. Differentially expressed genes (DEGs) were defined using DESeq2 v1.36.0 with an adjusted P-value < 0.05 and |fold change| > 1.5 (28). Gene Ontology (GO) terms highlighted in significant DEGs were accessed using ClusterProfiler v4.4. PCA plots, Volcano plots, bar plots, box plots, dot plots, and heatmaps were generated using ggplot2 v3.3.6 and ComplexHeatmap v2.13.1. All statistical analyses and visualizations were performed in R v4.2.1 and the R Studio environment (29). Biological Process (BP) terms were visualized using the emapplot function from the ClusterProfiler package to show correlations between them. Each dot represents significantly representative BP terms, and the size of each dot reflects the number of genes associated with the corresponding BP term that significantly increased or decreased in expression. The color of each dot in the visualization indicates the corrected p-value of the corresponding BP terms. The distance between dots reflects the calculated similarity or dissimilarity between BP terms, considering the gene content associated with each term. Closer dots indicate higher similarity, while dots further apart suggest greater dissimilarity. BP terms are grouped based on the calculated distances. Terms with higher similarity are grouped together, forming clusters or groups.

### 2.10 Statistical analysis

The experiments were repeated three times independently. We performed all statistical analyses with R, version 3.6.3 (30). The protection of immune priming, reflected by the frequency of living animals following a challenge at the LD_50_, was tested with a global mixed-effects Cox proportional hazard regression model (*coxme* packages) (30). Comparisons on survival between two conditions is presented as a hazard ratio (HR) that scores survival rate of a test group against survival in a referent group. A HR is reported with its 95% confidence interval and a Wald test p-value with Benjamini-Hochberg correction for multiple comparisons reporting whether the HR significantly deviates from 1. For hemocyte count and analysis, Student’s t-test was used to compare differences between groups in flow cytometry results. The experiment was repeated three times. Error bars indicate Mean ± SEM. *P < 0.05 (t-test). NS indicates not significant.

### 2.11 Data accessibility

The datasets generated and analyzed during this study have been uploaded to the GEO database (GSE266080).

## 3 Results

### 3.1 Comparative analysis of survival rates and hemocytes responses in group A-1 and B-1

**Figure 2:**
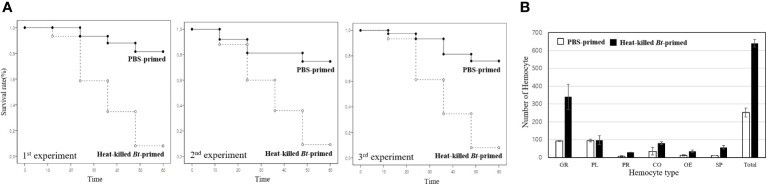
Kaplan-Meier survival analysis and hemocyte distribution between the PBS-primed group and the heat-killed *Bt*-primed group. **(A)** For comparative analysis of survival rates, groups were initially injected with PBS buffer and heat-killed *Bt*, respectively. After 2 days, all groups were injected with live *Bt.* Survival rate in the PBS-primed group was less than ~10%. However, in the heat-killed *Bt*-primed group, an average of 58 crickets survived after 60h, resulting in a ~80% survival rate. The higher survival rate observed in the heat-killed *Bt*-primed group compared to the PBS-primed group is attributed to the potential induction or maintenance of immune activation by the initial injection of heat-killed *Bt*. **(B)** After the second live *Bt* injection, hemocyte types and numbers were examined in each group. The PBS-primed group displayed an average of ~250/10µL hemolymph after 12h, while the heat-killed *Bt*-primed group exhibited an average of ~650/10µL hemolymph. Specifically, the PBS-primed group had an average of ~90 granulocytes, while the heat-killed *Bt*-primed group had ~330 granulocytes (~30 granulocytes/10µL hemolymph in untreated cricket). Plasmatocytes remained consistent (~100 in both groups), prohemocytes increased from ~8/10µL in the PBS-primed group to ~30/10µL in the heat-killed *Bt*-primed group. Coagulocytes rose from ~30/10µL in the PBS-primed group to ~80/10µL in the heat-killed *Bt*-primed group, oenocytoids increased from ~13/10µL in the PBS-primed group to ~40/10µL in the heat-killed *Bt*-primed group, and spherulocytes went from ~40/10µL in the PBS-primed group to ~60/10µL in the heat-killed *Bt*-primed group.

For the purpose of conducting a comparative analysis of survival rates, two groups were initially administered PBS buffer and heat-killed *Bacillus thuringiensis* (*Bt*), respectively. Following a 2-day interval, all groups were subsequently exposed to live *Bt* (**Figure 2A**; Conducted in triplicate with 75 crickets per experiment). All crickets were of nearly identical age (24 hours post-adult molting), exhibited good health (active movement and absence of infection), possessed an average size of approximately 25 ± 5mm per cricket, and an average weight of approximately 1.1 ± 0.5g per cricket.

In the PBS-primed group, crickets showed increased mortality post-live *Bt* injection, with survival rates dropping to less than 10% beyond 60 hours. The data indicates a noteworthy decline in survival rates over time, with a substantial proportion of crickets failing to survive beyond 60 hours. In contrast, the heat-killed *Bt*-primed group exhibited a remarkable 58 crickets surviving after 60 hours, yielding an approximate 80% survival rate. The higher survival rate observed in the heat-killed *Bt*-primed group is attributed to the potential induction or maintenance of immune activation following the initial injection of heat-killed *Bt*. This priming effect is believed to contribute to an augmented and swift immune response upon subsequent exposure to live *Bt*.

After the injection of live *Bt*, hemocyte types and numbers were examined in each group (**Figures 2B**). In untreated crickets, the average hemocyte count is ~150/10µL hemolymph. The PBS-primed group displayed an average of ~250/10µL hemolymph after 12h, while the heat-killed *Bt*-primed group exhibited an average of ~650/10µL hemolymph (**Figure 2B**). Specifically, the PBS-primed group had an average of ~90 granulocytes, while the heat-killed *Bt*-primed group had ~330 granulocytes (**Figure 2B**). There were no significant differences in plasmatocytes. In the heat-killed *Bt*-primed group, elevated levels of prohemocytes, coagulocytes, and spherulocytes were observed compared to the PBS-primed group (**Figure 2B**). These findings suggest that the initial injection of heat-killed *Bt* may have induced or sustained cellular immune activation, leading to elevated hemocyte counts, particularly granulocytes.

### 3.2 Correlating between granulocytes immune activation and enhanced survival: insight from morphological and functional analyses

**Figure 3:**
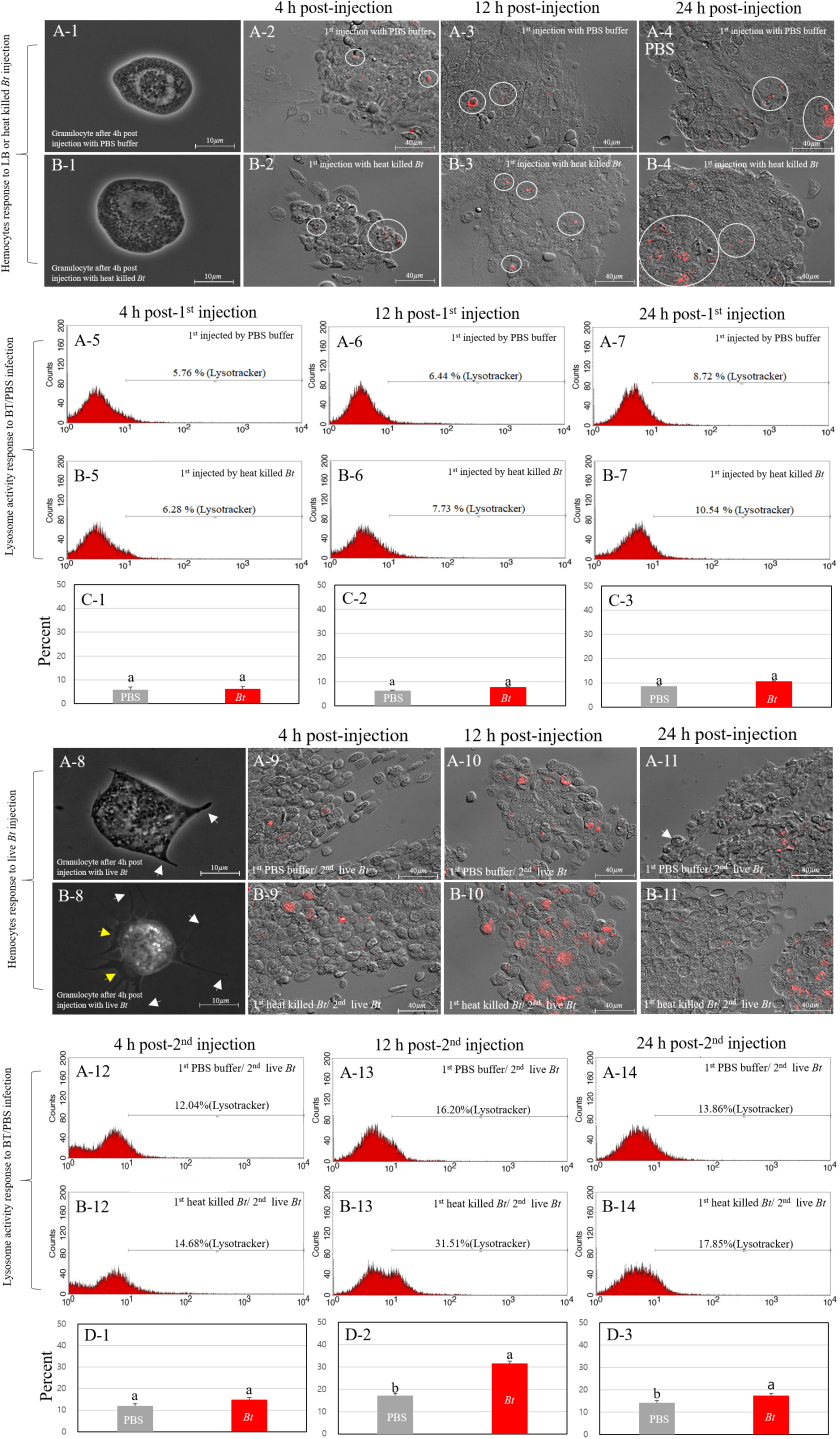
Immune activation dynamics in granulocytes. We explored the relationship between the elevated survival rate in group B-1 and the granulocyte immune activation level. Cricket granulocytes, when exposed to pathogens, undergo rapid morphological changes, forming pod structures (indicated by white yellows) and extracellular traps (ECTs indicated by yellow arrows) and engaging in phagocytosis, encapsulation, and nodulation. Fluorescence microscopy and fluorescence-activated cell sorting (FACS) were employed to observe ECTs formation and lysosome activation. The immune activation of granulocytes in groups **(A)** (PBS injection) and **(B)** (heat-killed *Bt* injection) was monitored over 4h, 12h, and 24h (Panels **A-1**~**A-7**, 3**B-1**~3**B-7**). Despite morphological changes in granulocyte size, statistical analysis of FACS results revealed no significant difference between group **(A)** (PBS) and group **(B)** (heat-killed *Bt*). Subsequent live *Bt* injections into groups A-1 and B-1 prompted morphological changes and lysosome activation over 4h, 12h, and 24h (Panels **A-8** ~**A-14**, **B-8**~**B-14**). Group A-1 showed larger granulocytes with pod formation, while group B-1 exhibited extensive pod and ECTs formation. Lysosome activity peaked at 12h in group A-1 and 24h in group B-1, suggesting prolonged immune response in the latter. The observed variations in lysosomal activity, granulocyte morphology, and behavior hint at potential differences in immune response and survival rates between groups A-1 and B-1. The flow cytometry analysis was repeated three times (Panels **C-1**~**C-3**, **D-1**~**D3**).

**Figure 4:**
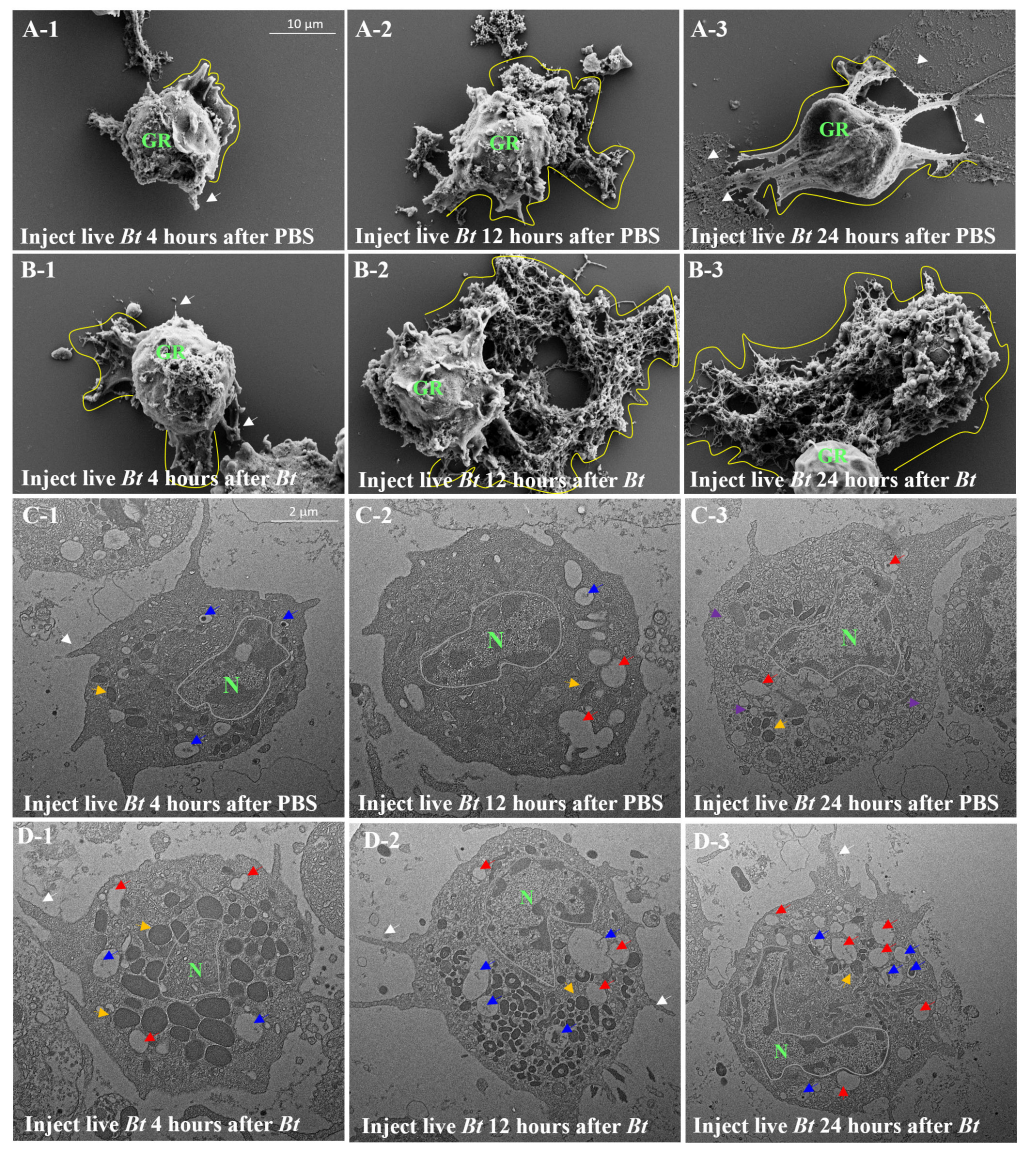
Comparison of granulocytes in group A and B using scanning electron microscopy (SEM) and transmission electron microscopy (TEM). Subsequent injection of live *Bt* into groups A and B led to morphological changes in granulocytes over 4, 12, and 24 hours (Panels **A-1** ~ **A-3**, **B-1** ~ **B-3**). Group **(A)** exhibited pods and ECTs formation, while group **(B)** displayed more rapid and prolonged immune activity. Transmission Electron Microscopy (TEM) was used to observe granulocyte cytoplasm (Panels **C-1**~**C-3**, **D-1**~**D-3**). Notable differences included increased black granules in Group **(B)** After live *Bt* reinjection, phagosomes containing live *Bt* were observed at 4 hours (Panels **D-1**~**D-3**; phagosomes indicated by blue arrows). Group **(B)** showed consistent lysosomes and black granules, maintaining a healthy state and active immune activities even after 24 hours, unlike group **(A)** The findings suggest a faster and prolonged cytological immune response in group B compared to group **(A)** Pods were observed on the cell membrane (GR means granulocytes and pods represented by white arrow). Yellow arrows represent black granules within granulocytes, notably produced in group **(B)** Red arrows denote large lysosomes in the cytoplasm of granulocytes. Purple arrows represent small-sized transparent granules in the cytoplasm of granulocytes in group A after 24 hours, indicating cytoplasmic vacuolization.

**Figure 5:**
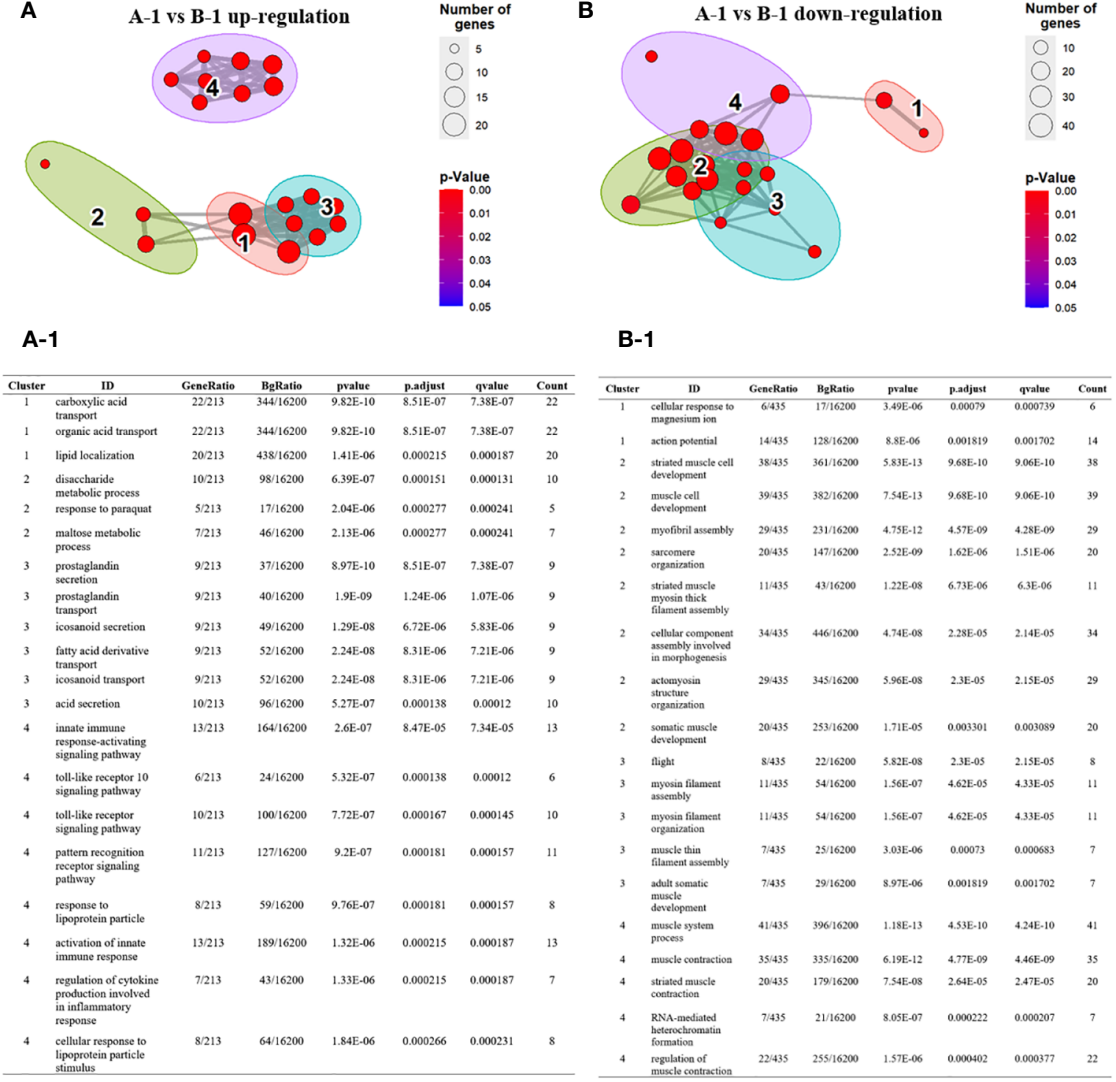
Over-representation (or enrichment) analysis of differentially expressed genes between group A-1 and group B-1. **(A, B)** Each node represents an enriched term and identified genes are explained in A-1 and B-1. Cutoff values included a *p*-value of <0.01, a minimum count of 3, and an enrichment factor of >1.5. GO biological processes, KEGG pathways, Reactome gene sets, CORUM complexes, and Canonical pathways from MsigDB were included in the search. Red nodes are colored by rations group A to group B **(A)** GO terms 1, 2, and 3 were grouped together based on similar functions. GO term 4 was associated with genes directly involved in insect innate immunity, such as Toll pathways, pattern recognition receptors (PRRs), and cytokines. (A-1) The identified genes are complex, encompassing diverse metabolic and signaling pathways, including those related to immunity. (**B**, B-1) Down-regulated GO terms in group B-1 were primarily associated with muscle-related genes.

We investigated the correlation between the higher survival rate of the heat-killed *Bt*-primed group and the morphological changes and degree of lysosome activation in granulocytes. After encountering pathogens, the cricket granulocytes exhibit rapid morphological changes in their cell membrane (forming extra-cellular traps (ECTs); fan- or pod-like structures) and eliminate pathogens through phagocytosis, encapsulation, and nodulation (18). Therefore, we observed the formation for ECTs in granulocytes and quantified activation of lysosomes following phagocytosis using the fluorescence microscopy and fluorescence-activated cell sorting (FACS) (**Figure 3**). First, we observed the degree of immune activation of granulocytes in group A (PBS buffer injection) and B (heat-killed *Bt* injection) over time (intervals of 4h, 12h, and 24h) (**Figures 3A-1~A-7, 3B-1~B-7**). **Figure 3A-1** illustrates the typical morphology of granulocytes 4 hours after the injection of PBS buffer. ECTs and pods (hair-like structures) related to immune activity were not observed on the cell membrane (**Figure 3A-1**). Most granulocytes in group B were observed to be relatively larger in size, but similar to granulocytes in group A, no other morphological changes were observed (**Figure 3B-1**). We collected hemocytes and examined lysosome activation at 4 hours, 12 hours, and 24 hours (**Figures 3A-2 ~ A-4, 3B-2 ~ B-4**). In groups A and B, lysosomal activity began to be observed in granulocytes at 4 h post-injection, and we did not observe any differences in lysosomal activity at 12- and 24-h post-injection. To quantify the red-fluorescent signals of groups A and B, hemocytes were analyzed by flow cytometry at 4h, 12h, and 48 h post-injection (**Figures 3A-5~A-7, 3B-5~B-7**). The average red fluorescent of group A was ~6.97% and that of group B was ~8.18%. The flow cytometry analysis was repeated three times (**Figures 3C-1~C-3**). Based on the results, it appears that despite observing changes in the size of granulocytes, the statistical analysis or interpretation of the FACS results for granulocyte immunity did not reveal a significant difference between group A (injected with PBS) and group B (injected with heat-killed *Bt*).

**Figure 6:**
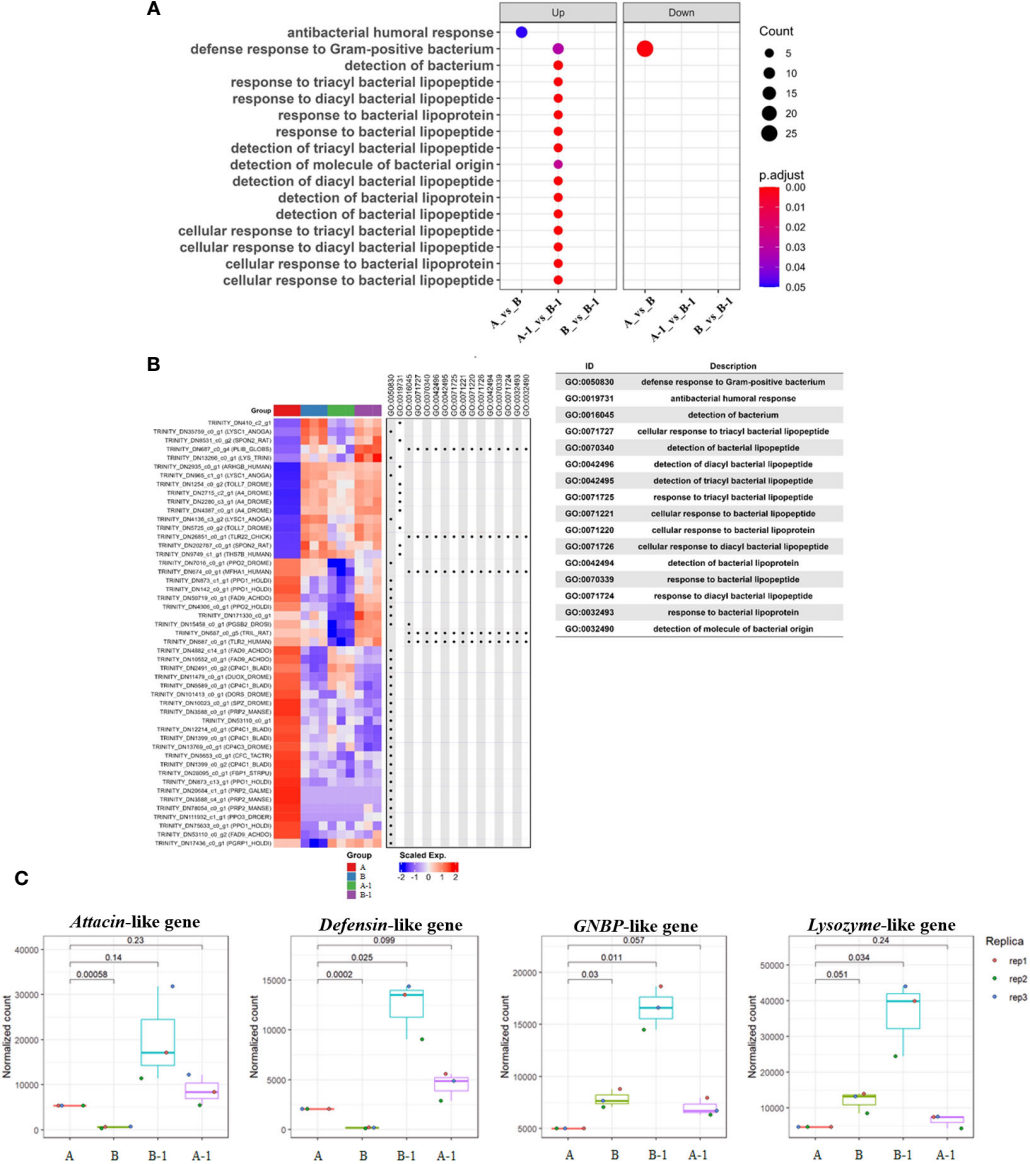
Dotplot and Heatmap analysis comparing the enrichment of multiple gene lists. Using a named list of gene clusters, the results were displayed as multiple columns with each one represents an enrichment result of a gene cluster. Utilizing ‘bacteria’ as a keyword in the Dotplot analysis, overexpressed GO terms in group B-1 compared to group A-1 were confirmed to be related to antibacterial humoral response, detection of bacterial lipopeptides, and cellular response to bacteria lipopeptides **(A)**. Heatmap analysis was performed to validate the aforementioned results, indicating that immune-related genes were significantly expressed in groups A-1, B, and B-1, excluding group A **(B)**. In addition, the Heatmap of gene set enrichment analysis (GSEA) of the hallmark gene sets in MsigDB database revealing the enrichment of antibacterial humoral response, detection and cellular response of bacterial lipopeptides. Notably, overexpressed humoral immune genes during bacterial invasion were identified. Normalization count analysis further confirmed high expression levels of *attacin*, *defensin*, *lysozyme*, and *GNBP*-like genes exclusively in group B-1 **(C)**.

**Figure 7:**
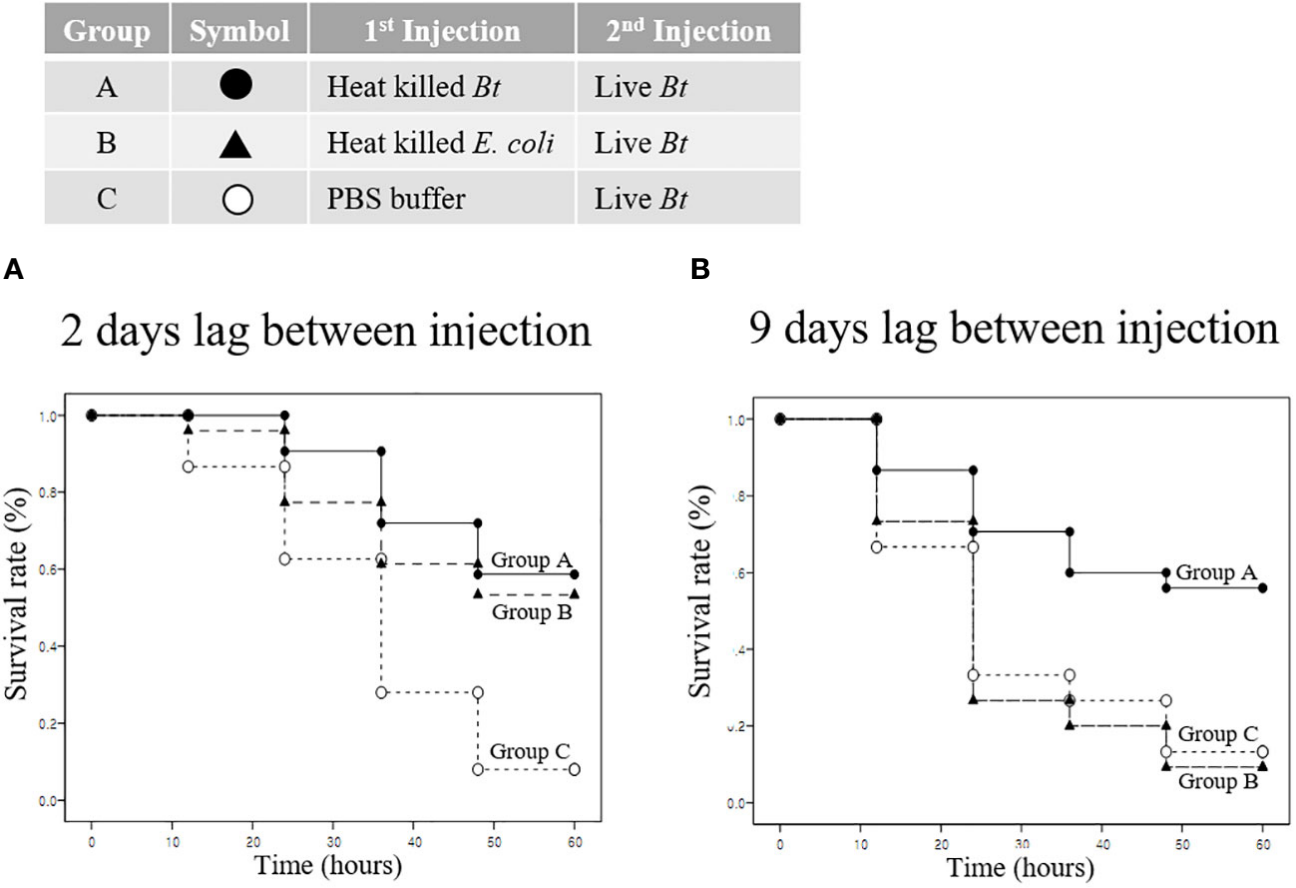
Priming effect and survival analysis according to pathogen type. **(A)** Short-term immune priming. Groups A, B, and C were initially injected with heat-killed *Bt*, heat-killed *E coli*, and PBS buffer, respectively. All groups were re-injected with live *Bt* to assess survival rates after two days. Control group C exhibited a low survival rate (~10%) after 60 hours, with limited mobility and signs of ill health. Group B showed an improved survival rate of approximately 50%, while group A demonstrated the highest survival rate (~60%) after 60 hours. Surviving crickets in groups A and B displayed high activity levels, engaging in mating and feeding activities. **(B)** Long-term immune priming. Nine days following the initial injection, a subsequent injection of Live *Bt* was administered to assess long-term survival rates. Both group C and group B displayed notably low survival rates, approximately 10% or less, after 60 hours, characterized by mostly motionless and unhealthy surviving crickets. Conversely, group A demonstrated a substantially higher survival rate, around 60%, with the majority of surviving crickets actively moving and in a healthy state after 60 hours.

Next, we performed a subsequent injection of live *Bt* into both groups A (previously injected with PBS) and B (previously injected with heat-killed *Bt*) two days after the initial injections (designated as group A-1 and B-1). To investigate morphological changes in granulocytes, we collected hemocytes at different time points (4 hours, 12 hours, and 24 hours) after the injection of live *Bt* (**Figures 3A-8~A-14, B-8~B-14**). At 4 h post-injection, most granulocytes in group A-1 appeared to be relatively larger in size and some pods began to form (**Figure 3A-8**; indicated by white arrows). On the other hand, granulocytes in group B-1 exhibited several very long pods (indicated by white arrow) and ECTs were also widely formed along the cell membrane, represented by yellow arrows (**Figure 3B-8**). To investigate the activation of lysosomes, we also collected hemocytes at different time points (4 hours, 12 hours, and 24 hours) after the injection of live *Bt*. At 4 h post-injection, lysosomal activation was observed in both groups A-1 and B-1. Subsequently, at 12 h post-injection, a higher level of lysosomal activation was observed in group B-1 compared to group A-1. By the 24 h post-injection, group A-1 exhibited reduced lysosomal activation, while group B-1 maintained sustained lysosomal activation (**Figures 3A-9~A-11, B-9~B-11**). To quantify the red-fluorescent signals of groups A-1 and B-1, hemocytes were analyzed by flow cytometry at 4h, 12h, and 48 h post-injection. In groups A-1 and B-1, lysosomal activity was first observed in granulocytes at 4 h post-injection and peaked at 12 hours (**Figures 3A-12, A-13, B-12, B-13**). At 24 h post-injection, lysosomal staining in the A-1 and B-1 groups was measured to be 13.84% and 17.85%, respectively.

### 3.3 Scanning electron microscopy and transmission electron microscopy analysis: morphological and cytoplasmic features revealing immune activity

After injection of PBS buffer or heat-killed *Bt*, the morphology of granulocytes at 4 hours is nearly identical to wild-type granulocytes (data not shown). Next, we performed a subsequent injection of live *Bt* into both the PBS-primed group and the heat-killed *Bt*-primed group two days after the initial injections. To investigate morphological changes, we collected granulocytes at different time points (4 hours, 12 hours, and 24 hours) after the injection of live *Bt* (**Figures 4A-1~A-3, B-1~B-3**). In the PBS-primed group, 4 hours after injection, pod structures (indicated by a white arrow) began to appear on the cell membrane, and ECTs started to form (**Figure 4A-1**; ECTs surrounded by yellow lines). After 12 hours, ECTs were extensively formed around the cell membrane of most granulocytes, reaching the peak of cellular immune activation (**Figure 4A-2**). After 24 hours, ECTs that had formed in granulocytes disappeared and were faintly observed (**Figure 4A-3**; ECTs losing their shape indicated by white arrows). As seen in **Figure 3A-11**, 24 hours after the live *Bt* injection, most granulocytes were observed to be unhealthy (**Figure 4A-3**). In the heat-killed *BT*-primed group, pod structures and ECTs had already begun to actively form on the granulocyte cell membrane after 4 hours (**Figure 4B-1**). By 12 hours after injection, the cell membranes of most granulocytes were completely transformed, and very large ECTs could be observed (**Figure 4B-2**). Additionally, in contrast to the PBS-primed group, most granulocytes were observed to maintain ECTs and continue immune activity after 24 hours (**Figure 4B-3**). The above results indicate that the heat-killed *BT*-primed group exhibited a faster cytological immune response to the re-injected activated pathogen (live *Bt*) and simultaneously, the duration of immunity was prolonged in the priming group.

Sequentially, we used TEM to observe the cytoplasmic features of granulocytes between groups (**Figures 4C-1 ~ 4C-3, 4D-1 ~ 4D-3**). The nucleus of the cell was labeled with a green ‘N’ and the nuclear membrane was outlined with a gray line around the nucleus. The yellow arrows indicate black granules of various sizes present in granulocytes, while the blue arrows are believed to represent phagosomes involved in the phagocytosis of *Bt*. Red arrows are considered to indicate early endosomes that occur after phagosome formation or lysosomes that have fused with the phagosome. First of all, the main difference observed between granulocytes in groups is the notable presence of black granules of various sizes in heat-killed *Bt*-primed group (**Figures 4D-1 ~ D-3**). These granules, recognized for containing various immune-related substances in mammalian white blood cells, were found in significant numbers within primed group (31). As indicated by the SEM results, morphological changes in the granulocytes of both groups were not observed solely after the initial injection (> 4 hours). However, a noteworthy finding was the significant production of granules within the cytoplasm of granulocytes in the heat-killed *Bt*-primed group (data not shown). In both groups, live *Bt* was reinjected and hemocytes were subsequently collected and photographed at intervals of 4 hours, 12 hours, and 24 hours, respectively. After 4 hours, phagosomes containing *Bt* fragments began to be observed in the granulocytes of both groups (**Figures 4C-1, D-1**; indicated by blue arrows). Additionally, large phagosomes were also observed, suggesting their growth through fusion with each other and potentially combining with lysosomes (indicated by red arrows). After 12 hours, large lysosomes were observable in the cytoplasm of granulocytes in both groups. Furthermore, a substantial number of black granules were consistently observed in the granulocytes of the heat-killed *Bt*-primed group (**Figure 4D-2**). After 24 hours, the cytoplasm of granulocytes in the PBS-primed group was observed to be filled with small-sized transparent granules, indicating cytoplasmic vacuolization (represented by purple arrows). Additionally, irregular disruption of the cell membrane was noted (**Figure 4C-3**). In contrast, the granulocyte cytoplasm of the heat-killed *Bt*-primed group consistently showed considerable-sized phagosomes (represented by blue arrows), large lysosome granules (represented by red arrow), and black granules of various sizes (represented by yellow arrows). Furthermore, pod-like structures were consistently observed in the granulocyte cytoplasm of the heat-killed *Bt*-primed (**Figure 4D-3**; represented by white arrow). In summary, the observed results indicate that granulocytes in the heat-killed *Bt*-primed group, when compared to PBS-primed group, maintained a healthy state and exhibited highly active immune activities even after 24 hours.

### 3.4 Comparative analysis of gene expression between groups

We conducted an analysis of the levels of gene activity to identify patterns and differences associated with the observed variations in survival rates and granulocyte activation among the experimental groups. To visualize the transcriptomic distances among the samples, dimension reduction using principal component analysis (PCA) was performed. In the 2-dimensional plot comprising the two most informative components, principal component 1 (PC1, explaining 47.9% of variance) and PC2 (explaining 12.9% of variance), the distances among the groups are clearly larger than the distances among samples within the same group (**Supplementary Figure S1A**). The samples in group A, injected only with PBS buffer, were positioned far from the samples in the other three groups. This indicates that the expression of genes in the samples injected with *Bt* differs significantly from those injected with PBS buffer only. To identify genes showing significant differences in their expressions between groups, we conducted differential gene expression (DEG) analysis and visualized the results using a volcano plot (**Supplementary Figures S1B-D**). Overall differences were observed in the gene expression patterns of each group, and these variances are believed to have influenced both the survival rate and the degree of cellular immune activation (**Supplementary Figure S1**).

Next, we visualized the relationship between the top four Gene Ontology (GO) terms based on significance. Specifically, in the comparison between group A-1 and group B-1, among the significant GO terms identified in group B-1, GO terms 1, 2, and 3 were grouped together based on similar functions. The identified genes are complex, encompassing diverse metabolic and signaling pathways, including those related to immunity. This underscores the importance of maintaining a balanced and regulated cellular environment to ensure homeostasis in energy metabolism and lipid localization (**Figures 5A-1**). Additionally, GO term 4 was associated with genes directly involved in insect innate immunity, such as Toll pathways, pattern recognition receptors (PRRs), and cytokines. Toll pathways, PRRs, and cytokines act as integral components of the insect immune system, working together to detect, signal, and coordinate immune responses against pathogens. Their intricate relationships form a robust defense mechanism critical for maintaining immune homeostasis. Down-regulated GO terms in group B-1 were primarily associated with muscle-related genes (**Figures 5B, B-1**). Comparing group A to group B, overexpressed GO terms in group B were associated with genes related to immune-related cell proliferation, differentiation, and developmental processes (**Supplementary Figures 2SA, 2SA-1**). Down-regulated GO terms were primarily linked to pigment and metabolism (**Supplementary Figures 2SB, 2SB-1**). When comparing group B to group B-1, the GO terms mainly identified were related to melanin synthesis. Down-regulated GO terms in this comparison were associated with muscle-related genes (**Supplementary Figures 2SC, 2SC-1, 2SD, 2SD-1**).

Utilizing ‘bacteria’ as a keyword in the Dotplot analysis, overexpressed GO terms in group B-1 compared to group A-1 were confirmed to be related to antibacterial humoral response, detection of bacterial lipopeptides, and cellular response to bacteria lipopeptides (**Figure 6A**). Notably, antibacterial humoral response (GO:0019731)-related genes were significantly upregulated in group A-1, B, and B-1 compared with group A (**Figure 6B**). Normalization count analysis further confirmed high expression levels of *attacin*, *defensin*, *lysozyme*, and *GNBP*-like genes exclusively in group B-1 (**Figure 6C**). Comparing the results, specifically between groups A and B, it was determined that the expression of genes inducing cell morphological changes commenced in group B. Additionally, when comparing A-1 and B-1, it was observed that group B-1 exhibited overexpression of various genes related to both humoral and cellular immunity.

### 3.5 Analysis of short-term and long-term immune priming effects

We analyzed the priming effect and survival when the pathogen used for immunity induction differed from the reinvading pathogen. Group A, B, and C were injected with heat-killed *Bt*, heat-killed *E*. *coli*, and PBS buffer, respectively. Subsequently, all groups were re-injected with live *Bt* to assess survival rates. Survival rates were evaluated for two- and nine-day intervals between injections. Two days later, in the control group C injected with live *Bt*, the survival rate was approximately 10% (**Figure 7A**). After 60 hours, the survival rate for groups A and B was approximately 60%, and the surviving crickets remained highly active and healthy (**Figure 7A**). Next, live *Bt* was injected 9 days after the first injection to examine survival rates among groups (**Figure 7B**). Surprisingly, while group C and B exhibited low survival rates of ~10% until 60 hours after injection, group A demonstrated a high survival rate of ~60% after 60 hours (**Figure 7B**). To confirm the results in **Figure 7**, the survival survey was replicated three times (**Supplementary Figure S3**). During short-term immune priming, there is an observed elevated immune response to both homologous and heterogeneous pathogens (indicating a cross-priming effect). However, it is presumed that crickets exhibit an enhanced immune response exclusively to the homologous pathogen used during long-term immune priming.

## 4 Discussion

Recently, scientists in the fields of entomology and immunology have addressed a variety of questions about how insects have evolved over time to fight off a variety of pathogens without having the complex adaptive immune systems that exist in vertebrate. Essentially, this raises the question of whether insects might possess mechanisms similar to immune priming or memory (11). A number of studies have centered around this question, and this study, initiated by these inquiries, particularly explores the relationship between cellular immune responses based on immune priming.

In the field of immunology, demonstrating the effectiveness of immune priming (short-term, long-term, cross-priming, etc.) in insects is a very challenging task. This involves complex tasks such as determining the period from priming to pathogen reinfection and setting the infection dose (13, 24). For example, if the interval between immune priming and the second infection is too short or too long, distinguishing the effects of immune priming against the same or different pathogens used during initial priming becomes complicated. Moreover, injecting small doses of the pathogen during immune priming makes it difficult to obtain a significant immune priming effect, whereas injecting large doses increases the mortality rate of insects due to secondary infections during poor health, complicating the interpretation of the results. The results of this study precisely established the optimal dose and duration of exposure to the pathogen through a variety of preliminary experiments. For example, considering that the adult lifespan of the experimental insect, the cricket, is 30 ± 5 days, the period from immune priming to live *Bt* injection was precisely determined (**Figures 2**, **7**). The 2-day group had a significantly higher survival rate compared to the PBS-injected group (**Figures 2**, **7**). These findings suggest a cross-priming effect, wherein the resistance of crickets to other pathogens is simultaneously enhanced when exposed to a single pathogen. Interestingly, the appropriately primed group (9 days) displayed an immune-boosting effect specifically against the same type of bacteria, while the cross-priming effect diminished. This phenomenon may be attributed to immune blood cells retaining bacterial cell fragments post-phagocytosis, with these fragments persisting in the hemolymph for some time. Upon re-exposure to the same specific bacteria, they induce a faster and stronger immune response (3). Furthermore, it can be hypothesized that specific pattern recognition receptors (PRRs) targeting allogeneic bacteria remain in the hemolymph for an extended period without degradation, thereby eliciting a rapid immune response upon re-invading allogenic bacteria (25–29). As depicted in **Figure 4D-3**, granulocytes were observed to consistently retain bacterial cell fragments. These retained fragments may persist in the hemolymph for some time, inducing a faster and more powerful immune response upon re-exposure to the same specific bacteria. In the future, delving into the specific molecular pathways engaged in preserving bacterial cell fragments by granulocytes will be indispensable.

We investigated the effect of immune priming in conjunction with the activation of immune cells. To date, there have been few studies that have tested the effects of immune priming with a focus on the detailed observation of immune cells. In insects, the immune priming effect linked to hemocytes has been reported in *Anopheles gambiae* (15). The immune-priming group exhibited a high survival rate accompanied by an increase in the number of hemocytes, suggesting the significance of augmented immune cells in pathogen removal and overall survival (10, 32). Our study similarly confirmed that in the heat-killed *Bt*-primed group, all six types of hemocytes increased. Among them, granulocytes, the principal immune cells in this insect, demonstrated the most significant increase. These results align with observations in vertebrate, where an overall increase in blood cells is associated with protection against pathogen infection (2, 33). Notably, an elevation in white blood cells has been reported to enhance the likelihood of survival. Additionally, detailed immune-morphological changes of granulocytes were also reported in this study. As illustrated in **Figures 2**, **3**, and **4**, the observed increase in granulocytes, coupled with their rapid morphological changes indicative of heightened phagocytic activity, led to the swift and efficient removal of re-invading pathogens, resulting in an enhanced survival rate compared to the control group.

The validation of immune priming in insects has primarily been explored in association with the swift production of antimicrobial peptides (AMPs). For instance, research has reported an increase in survival rates upon reinfection with specific pathogens in *Drosophila melanogaster*, *Galleria mellonella*, *Bombus terrestris*, and *Anopheles gambiae* after immune priming (34–37). As evident in **Figure 6**, we also confirmed the overexpression of certain AMPs (*attacin*, *defensin*, and *lysozyme*) exclusively in the heat-killed *Bt*-primed group subjected to immune priming with pathogens. This suggests that the rapid immune response of granulocytes, coupled with the swift overexpression of antibiotic proteins, contributed to the increased survival rate.

While modeled insects like *D. melanogaster* and *A. gambiae* offer the advantage of conducting experiments under controlled laboratory conditions across multiple generations, yielding consistent and reproducible results, their immune responses to various pathogens may exhibit slight variations compared to insects in their natural habitats. In the wild, insects have endured prolonged exposure to diverse infectious agents, developing robust defenses against invading pathogens. Although the fundamental immune system is remarkably similar across all insects, individuals or groups may manifest varying strengths in immune responses depending on the environmental conditions of their habitat (38). Crickets, thriving in highly humid environments, are exposed to a diverse array of pathogens. It can be inferred that insects in such habitats have evolved while constantly contending with the persistent threat of pathogen infection (18). We conducted our study by collecting and raising adult crickets directly in their natural environment, obtaining results from 2^nd^ to 3^rd^ generation crickets. Therefore, we posit that the notably active immune activity of granulocytes observed in our study is readily observable. However, crickets, unlike other model insects, lack reported genomic reference genes, posing a limitation that hindered the precise analysis of many specifically overexpressed genes.

In summary, our study revealed that prolonged immune activation, characterized by heightened granulocyte activity, phagocytosis, and extracellular trap formation, contributes to enhanced survival rates. Gene expression analysis supports these findings, indicating the differential regulation of immune-related genes. In addition, crickets manifest a cellular immune response linked to both short-term and long-term priming effects. Short-term priming induces a cross-priming effect, whereas long-term priming results in a diminished cross-priming effect, emphasizing a swift immune response primarily directed at the same type of bacteria.

## Data availability statement

The datasets presented in this study can be found in online repositories. The names of the repository/repositories and accession number(s) can be found in the article/**Supplementary Material**.

## Ethics statement

The manuscript presents research on animals that do not require ethical approval for their study.

## Author contributions

YC: Data curation, Formal Analysis, Investigation, Validation, Visualization, Writing – original draft, Methodology. SC: Data curation, Formal Analysis, Investigation, Validation, Visualization, Writing – original draft, Conceptualization, Funding acquisition, Supervision, Writing – review & editing.

## Supplementary material

Supplementary Figure 1 Principal component analysis (PCA) and volcano plots of differentially expressed genes (DEGs). Group A was injected with only PBS buffer. Group B was injected with heat-killed *Bt*. Group A-1 was injected with live *Bt* after 2 days of PBS buffer injection. Group B-1 was injected with live *Bt* after two days of heat-killed *Bt* injection. **(A)** PCA of all extracts. The volcano plot of A group vs. B group **(B)**, A-1 group vs. B-1 group **(C)**, and B group vs. B-1 group **(D)**. Groups A-1, B, and B-1 were distinguished from the control group (group A), indicating that the injected material played a crucial role in transcriptome formation. Simultaneously, groups A-1, B, and B-1 formed distinct clusters, suggesting that the injected substances were the primary factors determining overall gene expression. In comparison to group B-1, group A exhibited significant expression differences in a total of 24,584 genes, with 12,149 up-regulated genes and 12,435 down-regulated genes. Group B-1, compared to group A-1, displayed expression differences in a total of 6,025 genes, with 2,569 up-regulated genes and 3,456 down-regulated genes. In group B-1, compared to B, a total of 5,402 gene expression differences were confirmed, with 2,829 up-regulated genes and 2,573 down-regulated genes. The aforementioned results illustrate variations in the expression patterns of all genes based on the immune-inducing substance, suggesting that these differences may have influenced the survival rate and the degree of cellular immune activation.

Supplementary Figure 2 Over-representation (or enrichment) analysis of differentially expressed genes between group A and B, as well as between group B and B-1. Each node represents an enriched term **(A, B, C, D)** and identifies genes are explained in A-1, B-1, C-1, and D-1. Cutoff values included a *p*-value of <0.01, a minimum count of 3, and an enrichment factor of >1.5. GO biological processes, KEGG pathways, Reactome gene sets, CORUM complexes, and canonical pathways from MsigDB were included in the search. Red nodes are colored by rations between group A and B, as well as between group B and B-1. Comparing group A to group B, overexpressed GO terms in group B were associated with genes related to immune-related cell proliferation, differentiation, and developmental processes (**A** and A-1). Down-regulated GO terms were primarily linked to pigment and metabolism (**B** and B-1). Comparing group B to group B-1, the GO terms mainly identified were related to melanin synthesis (**C** and C-1). Down-regulated GO terms in this comparison were associated with muscle-related genes (**D** and D-1).

Supplementary Figure 3 Priming effect and survival analysis according to pathogen type. To confirm the results in , the survival survey was replicated three times. Crickets demonstrate both short-term and long-term immune priming effects.

## References

[1] Yamauchi T Moroishi T. Hippo pathway in mammalian adaptive immune system. Cells. (2019) 30:398. doi: 10.3390/cells8050398 PMC6563119 31052239

[2] Marshall JS Warrington R Watson W Kim HL. An introduction to immunology and immunopathology. Allergy Asthma Clin Immunol. (2018) 12:49. doi: 10.1186/s13223-018-0278-1 PMC6156898 30263032

[3] Hillyer JF. Insect immunology and hematopoiesis. Dev Comp Immunol. (2016) 58:102–18. doi: 10.1016/j.dci.2015.12.006 PMC4775421 26695127

[4] Tsuzuki S Matsumoto H Furihata S Ryuda M Tanaka H Sung EJ. Switching between humoral and cellular immune responses in *Drosophila* is guided by the cytokine GBP. Nat Commun. (2014) 5:4628. doi: 10.1038/ncomms5628 25130174 PMC5755975

[5] Lavine MD Strand MR. Insect hemocytes and their role in immunity. Insect Biochem Mol Biol. (2002) 32:1295–309. doi: 10.1016/S0965-1748(02)00092-9 12225920

[6] Lemaitre B Hoffmann J. The host defense of Drosophila melanogaster. Annu Rev Immunol. (2007) 25:697–743. doi: 10.1146/annurev.immunol.25.022106.141615 17201680

[7] Brinkmann V Reichard U Goosmann C Fauler B Uhlemann Y Weiss DS. Neutrophil extracellular traps kill bacteria. Science. (2004) 303:1532–5. doi: 10.1126/science.1092385 15001782

[8] Robb C Dyrynda E Gray R Rossi A Smith V. Invertebrate extracellular phagocyte traps show that chromatin is an ancient defence weapon. Nat Commun. (2014) 5:4627. doi: 10.1038/ncomms5627 25115909 PMC4143918

[9] Chen RY Keddie BA. *Galleria mellonella* (Lepidoptera: Pyralidae) hemocytes release extracellular traps that confer protection against bacterial infection in the hemocoel. J Insect Sci. (2021) 21:17. doi: 10.1093/jisesa/ieab092 PMC8643984 34865034

[10] Cooper D Eleftherianos I. Memory and specificity in the insect immune system: current perspectives and future challenges. Frontier Immunol. (2017) 8:539. doi: 10.3389/fimmu.2017.00539 PMC5422463 28536580

[11] Lanz-Mendoza H Garduño JC. Insect Innate Immune Memory. In: Cooper EL, editor. Advances in comparative immunology. Springer International Publishing (2018). p. 193–211.

[12] Netea MG Domínguez-Andrés J Barreiro LB Chavakis T Divangahi M Fuchs E. Defining trained immunity and its role in health and disease. Nat Rev Immunol. (2020) 20:375–88. doi: 10.1038/s41577-020-0285-6 PMC7186935 32132681

[13] Gomes FM Silva M Molina-Cruz A Barillas-Mury C. Molecular mechanisms of insect immune memory and pathogen transmission. PloS Pathog. (2022) 18:e1010939. doi: 10.1371/journal.ppat.1010939 36520682 PMC9754258

[14] Cabrera K Hoard DS Gibson O Martinez DI Wunderlich Z. *Drosophila* immune priming to *Enterococcus faecalis* relies on immune tolerance rather than resistance. PloS Pathog. (2023) 19:e101156. doi: 10.1371/journal.ppat.1011567 PMC10446173 37566589

[15] Rodrigues J Brayner FA Alves LC Dixit R Barillas-Mury C. Hemocyte differentiation mediates innate immune memory in. Anopheles Gambiae mosquitoes Sci. (2020) 329:1353–5. doi: 10.1126/science.1190689 PMC3510677 20829487

[16] Kulkarni S Kurapati S Bogunovic M. Neuro-innate immune interactions in gut mucosal immunity. Curr Opin Immunol. (2021) 68:64–71. doi: 10.1016/j.coi.2020.09.007 33130386 PMC11095515

[17] Melillo D Marino R Italiani P Boraschi D. Innate immune memory in vertebrate metazoans: A critical appraisal. Front Immunol. (2018) 9:2018. doi: 10.3389/fimmu.2018.01915 30186286 PMC6113390

[18] Ferro K Peuß R Yang W Rosenstiel P Schulenburg H Kurtz J. Experimental evolution of immunological specificity. Proc Natl Acad Sci. (2019) 116:20598–604. doi: 10.1073/pnas.1904828116 PMC6789748 31548373

[19] Ng TH Harrison MC Scharsack JP Kurtz J. Disentangling specific and unspecific components of innate immune memory in a copepod-tapeworm system. Front Immunol. (2024) 15:1307477. doi: 10.3389/fimmu.2024.1307477 38348037 PMC10859752

[20] Wang W Li Y Fan S Lian X Cao W Song X. The elevated expressions of anti-lipopolysaccharide factors Aater priming stimulation confer lastingly humoral protection in crab *Eriocheir sinensis* . Front Immunol. (2021) 12:757434. doi: 10.3389/fimmu.2021.757434 PMC8692716 34956187

[21] Kwon H Bang K Cho S. Characterization of the Hemocytes in Larvae of *Protaetia brevitarsis seulensis*: Involvement of Granulocyte-Mediated Phagocytosis. PloS One. (2014) 9:e103620. doi: 10.1371/journal.pone.0103620 25083702 PMC4118905

[22] Hwang S Bang K Lee J Cho S. Hemocytes from larvae of the Japanese rhinoceros beetle *allomyrina dichotoma* (Linnaeus) (Coleoptera: scarabaeidae) and the cellular immune response to microorganisms. PloS One. (2015) 10:e0128519. doi: 10.1371/journal.pone.0128519 26030396 PMC4452365

[23] Lee J Hwang S Cho S. Immune tolerance to an intestine-adapted bacteria, *Chryseobacterium* sp., injected into the hemocoel of *Protaetia brevitarsis seulensis* . Sci Rep. (2016) 6:1–14. doi: 10.1038/srep31722 27530146 PMC4987663

[24] Cho Y Cho S. Hemocyte-hemocyte adhesion by granulocytes is associated with cellular immunity in the cricket, Gryllus bimaculatus. Sci Rep. (2019) 9:18066. doi: 10.1038/s41598-019-54484-5 31792279 PMC6889498

[25] Grabherr M Haas B Yassour M Levin J Thompson D Amit I. Full-length transcriptome assembly from RNA-Seq data without a reference genome. Nat Biotechnol. (2011) 29:644–52. doi: 10.1038/nbt.1883 PMC3571712 21572440

[26] Bryant M Felice M Briscoe T. (2017). Automatic annotation and evaluation of error types for grammatical error correction, in: Proceedings of the 55th Annual Meeting of the Association for Computational Linguistics (Volume 1: Long Papers), 793–805. https://aclanthology.org/P17-1074

[27] Patro R Duggal G Love M Irizarry R Kingsford C. Salmon provides fast and bias-aware quantification of transcript expression. Nat Methods. (2017) 14:417–9. doi: 10.1038/nmeth.4197 PMC5600148 28263959

[28] Love MI Huber W Anders S. Moderated estimation of fold change and dispersion for RNA-seq data with DESeq2. Genome Biol. (2014) 5:550. doi: 10.1101/002832 PMC4302049 25516281

[29] R Core Team. R: A language and environment for statistical computing. R Foundation for Statistical Computing (2020). Available at: https://www.R-project.org/.

[30] Therneau TM Lumley T. coxme: Mixed Effects Cox Models. R package version 2.2–16 (2022). Available at: https://CRAN.R-project.org/package=coxme.

[31] Sheshachalam A Srivastava N Mitchell T Lacy P Eitzen G. Granule protein processing and regulated secretion in neutrophils. Front Immunol. (2014) 5:448. doi: 10.3389/fimmu.2014.00448 25285096 PMC4168738

[32] Garcia-Valtanen P Guzman-Genuino RM Williams DL Hayball JD Diener KR. Evaluation of trained immunity by β-1, 3 (d)-glucan on murine monocytes in *vitro* and duration of response in *vivo* . Immunol Cell Biol. (2017) 95:601–10. doi: 10.1038/icb.2017.13 PMC5550561 28228641

[33] Sadd BM Schmid-Hempel P. Insect immunity shows specificity in protection upon secondary pathogen exposure. Curr Biol. (2006) 16:1206–10. doi: 10.1016/j.cub.2006.04.047 16782011

[34] Pham LN Dionne MS Shirasu-Hiza M Schneider DS. A specific primed immune response in *Drosophila* is dependent on phagocytes. PloS Pathol. (2007) 3:e26. doi: 10.1371/journal.ppat.0030026 PMC1817657 17352533

[35] Brown LD Shapiro LLM Thompson GA Estévez-Lao TY Hillyer JF. Transstadial immune activation in a mosquito: Adults that emerge from infected larvae have stronger antibacterial activity in their hemocoel yet increased susceptibility to malaria infection. Ecol Evol. (2019) 9:6082–95. doi: 10.1002/ece3.5192 PMC6540708 31161020

[36] Zhang QY Yan ZB Meng YM Hong XY Shao G Ma JJ. Antimicrobial peptides: mechanism of action, activity and clinical potential. Military Med Res. (2021) 8:48. doi: 10.1186/s40779-021-00343-2 PMC8425997 34496967

[37] Gomes FM Tyner MDW Barletta ABF Saha B Yenkoidiok-Douti L Canepa GE. Double peroxidase and histone acetyltransferase AgTip60 maintain innate immune memory in primed mosquitoes. PNAS. (2021) 118:e2114242118. doi: 10.1073/pnas.2114242118 34711682 PMC8612215

[38] Shikano I Cory JS. Impact of environmental variation on host performance differs with pathogen identity: Implications for host-pathogen interactions in a changing climate. Sci Rep. (2015) 5:15351. doi: 10.1038/srep15351 26477393 PMC4609993

