# Supplementary figures and images for: Granulocyte dynamics: a key player in the immune priming effects of crickets
Source: Front Immunol. 2024 May 17;15:1383498. doi: 10.3389/fimmu.2024.1383498 (PMC11140058; doi:10.3389/fimmu.2024.1383498)

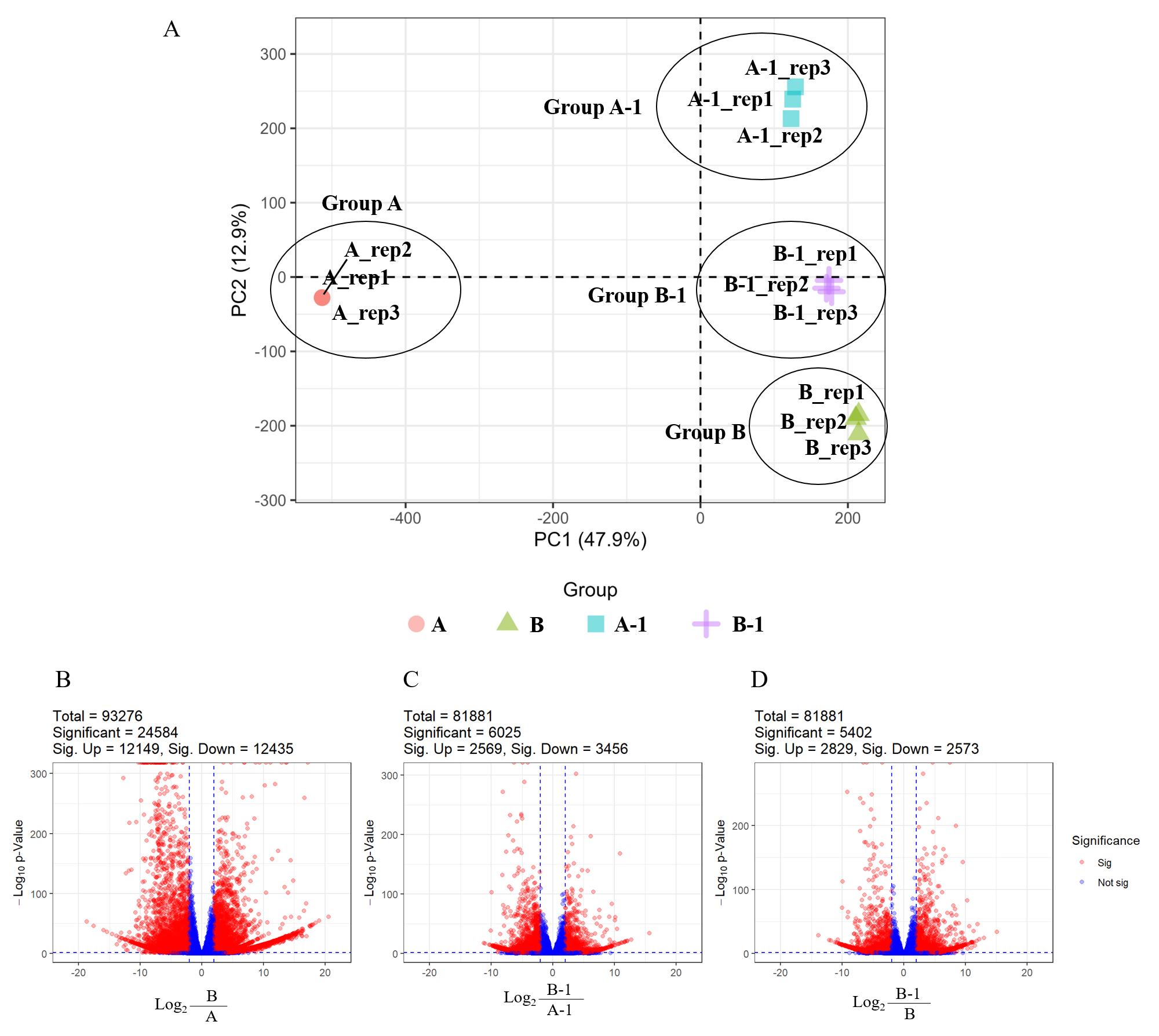

Supplement: Supplementary Figure 1 — Principal component analysis (PCA) and volcano plots of differentially expressed genes (DEGs). Group A was injected with only PBS buffer. Group B was injected with heat-killed Bt. Group A-1 was injected with live Bt after 2 days of PBS buffer injection. Group B-1 was injected with live Bt after two days of heat-killed Bt injection. (A) PCA of all extracts. The volcano plot of A group vs. B group (B), A-1 group vs. B-1 group (C), and B group vs. B-1 group (D). Groups A-1, B, and B-1 were distinguished from the control group (group A), indicating that the injected material played a crucial role in transcriptome formation. Simultaneously, groups A-1, B, and B-1 formed distinct clusters, suggesting that the injected substances were the primary factors determining overall gene expression. In comparison to group B-1, group A exhibited significant expression differences in a total of 24,584 genes, with 12,149 up-regulated genes and 12,435 down-regulated genes. Group B-1, compared to group A-1, displayed expression differences in a total of 6,025 genes, with 2,569 up-regulated genes and 3,456 down-regulated genes. In group B-1, compared to B, a total of 5,402 gene expression differences were confirmed, with 2,829 up-regulated genes and 2,573 down-regulated genes. The aforementioned results illustrate variations in the expression patterns of all genes based on the immune-inducing substance, suggesting that these differences may have influenced the survival rate and the degree of cellular immune activation. [file Image_1.png]

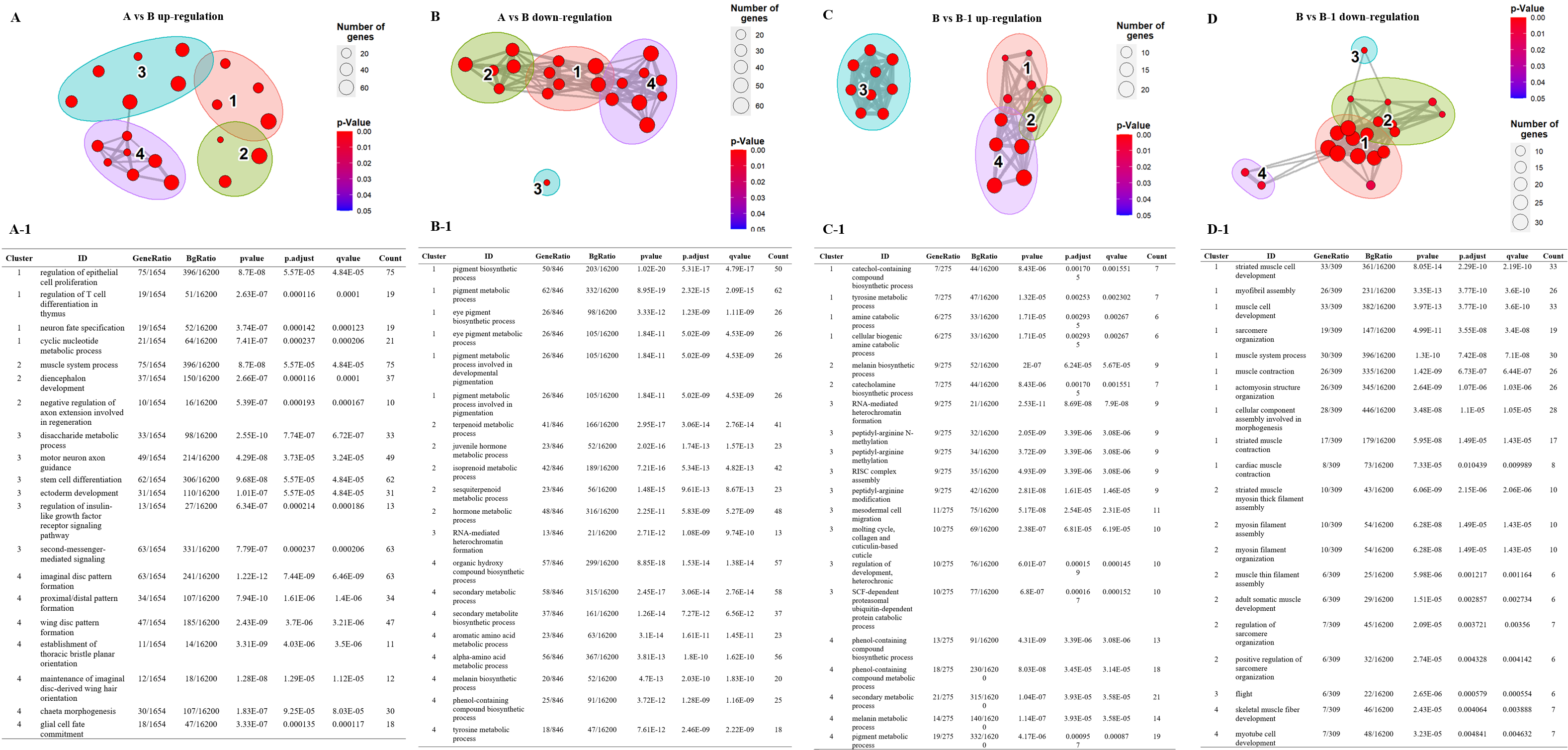

Supplement: Supplementary Figure 2 — Over-representation (or enrichment) analysis of differentially expressed genes between group A and B, as well as between group B and B-1. Each node represents an enriched term (A, B, C, D) and identifies genes are explained in A-1, B-1, C-1, and D-1. Cutoff values included a p-value of <0.01, a minimum count of 3, and an enrichment factor of >1.5. GO biological processes, KEGG pathways, Reactome gene sets, CORUM complexes, and canonical pathways from MsigDB were included in the search. Red nodes are colored by rations between group A and B, as well as between group B and B-1. Comparing group A to group B, overexpressed GO terms in group B were associated with genes related to immune-related cell proliferation, differentiation, and developmental processes (A and A-1). Down-regulated GO terms were primarily linked to pigment and metabolism (B and B-1). Comparing group B to group B-1, the GO terms mainly identified were related to melanin synthesis (C and C-1). Down-regulated GO terms in this comparison were associated with muscle-related genes (D and D-1). [file Image_2.png]

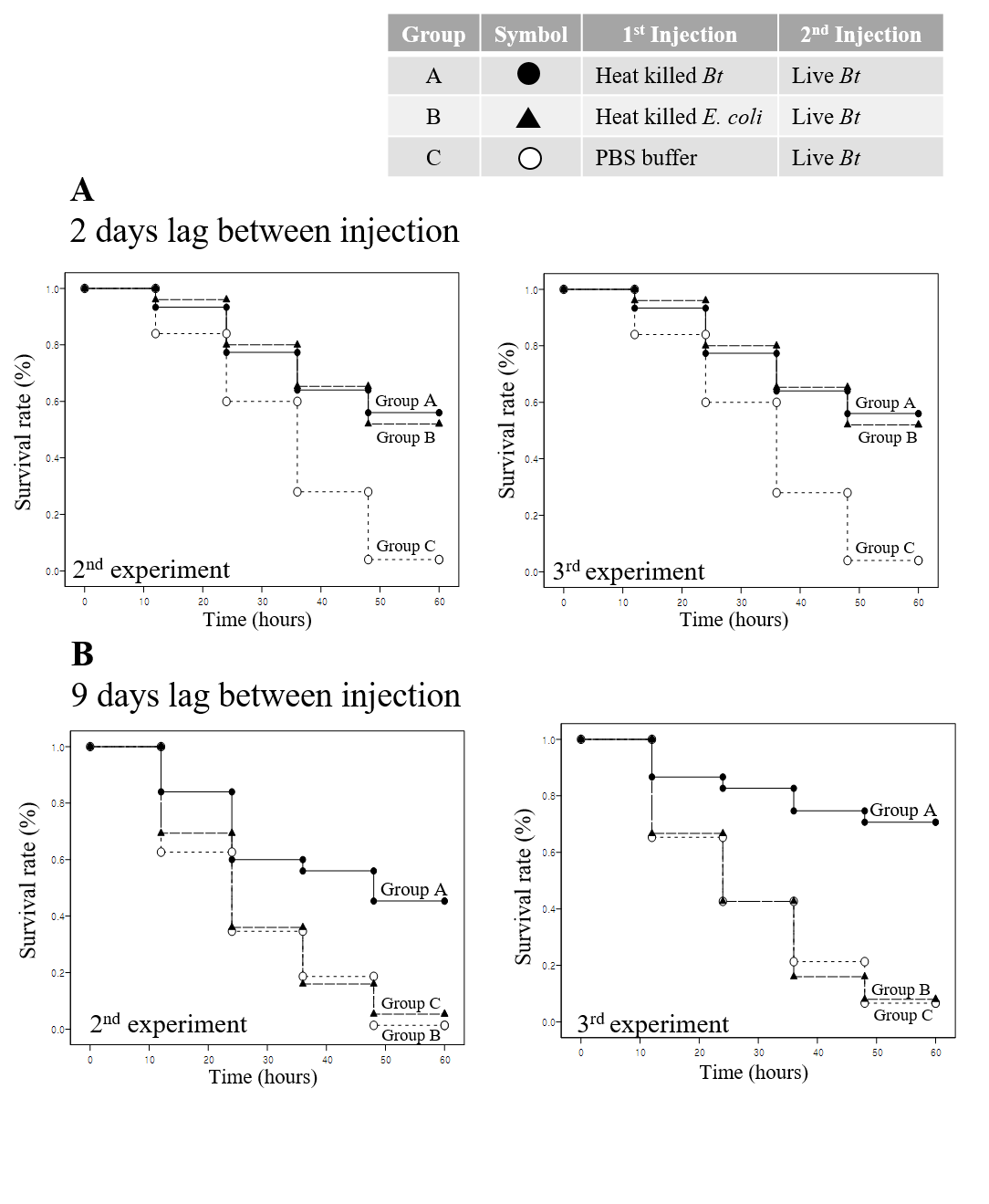

Supplement: Supplementary Figure 3 — Priming effect and survival analysis according to pathogen type. To confirm the results in , the survival survey was replicated three times. Crickets demonstrate both short-term and long-term immune priming effects. [file Image_3.tif]
