# Supplementary material for: Granulocyte dynamics: a key player in the immune priming effects of crickets
Source: Front Immunol. 2024 May 17;15:1383498. doi: 10.3389/fimmu.2024.1383498 (PMC11140058; doi:10.3389/fimmu.2024.1383498)
Supplement: Supplementary file 4 [file DataSheet_1.docx]

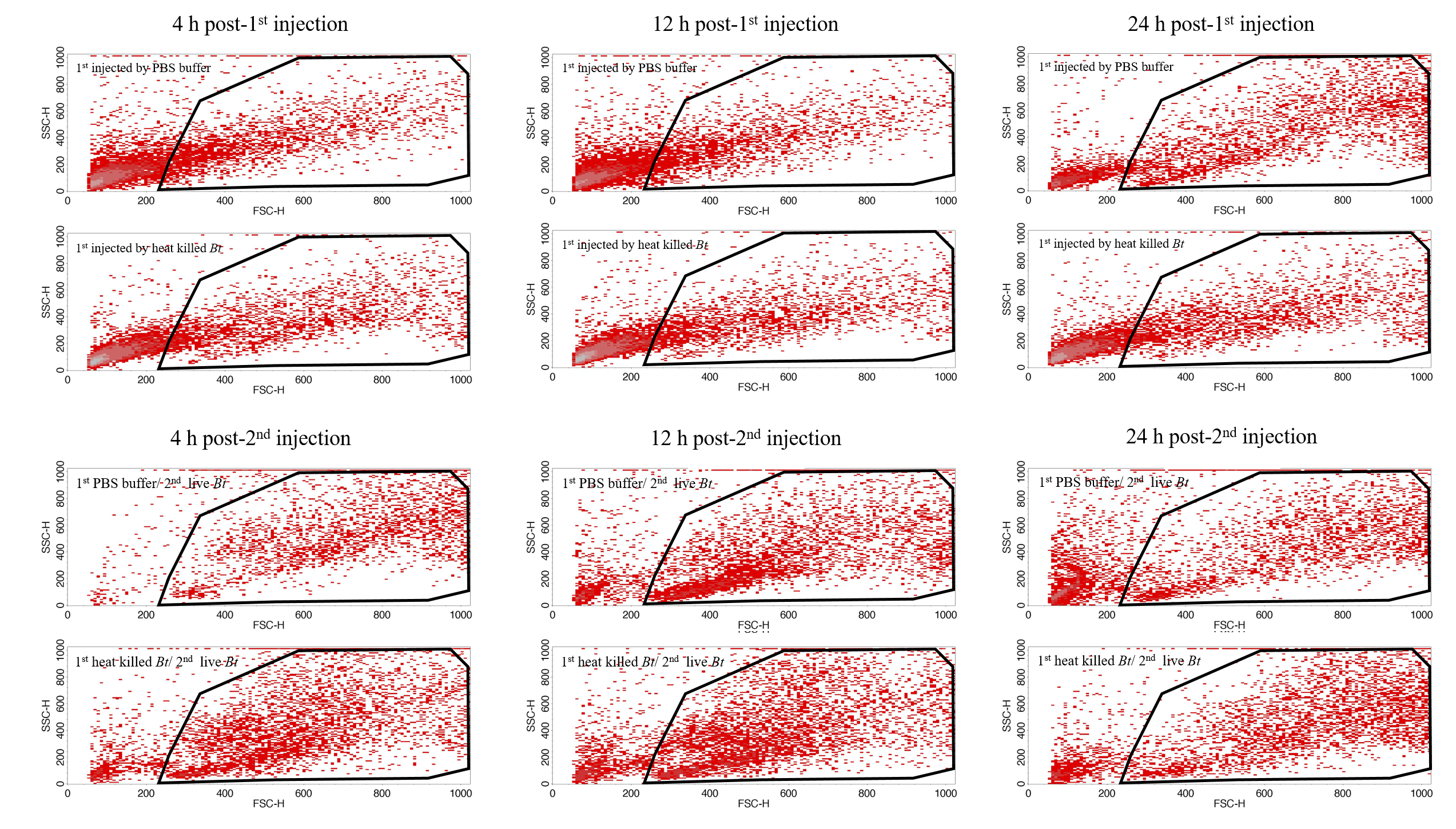


Fig 1S. Gating strategy for flow cytometry analysis for Fig. 3. In this sample gating, cells were gated in an SSC-H and FSC-H dot plot to select 6 types of hemocytes. Cricket's immune cells, granulocytes, differ from mammalian T cells and B cells in that they do not aggregate (1). We have defined a range encompassing six types of hemocytes. Lysotracker is capable of assessing immune activation levels by selectively staining the lysosomes of immune-activated granulocytes (2).

Reference

1. Mao F, Wong NK , Lin Y, Zhang X, Liu K, Huang M, Xu D, Xiang Z, Li J, Zhang Y, Yu Z. Transcriptomic Evidence Reveals the Molecular Basis for Functional Differentiation of Hemocytes in a Marine Invertebrate, *Crassostrea gigas* *Frontiers in Immunology* (2020) 11.
2. Cho Y, Cho S. Hemocyte-hemocyte adhesion by granulocytes is associated with cellular immunity in the cricket, *Gryllus bimaculatus. Scientific Reports* (2019) 9:18066.
